# A Tale of Two Organs: The Liver–Lung Axis in Pulmonary Disease

**DOI:** 10.1002/cph4.70076

**Published:** 2025-12

**Authors:** Arun Jose, Hilary M. DuBrock, Navneet Singh, Jay Kolls, Michael Krowka, Rudolf K. F. Oliveira, Sarah Raevens, Adriano R. Tonelli, Kathryn del Valle, Corey E. Ventetuolo, Usha Raj

**Affiliations:** 1 University of Cincinnati, Cincinnati, Ohio, USA; 2 Mayo Clinic College of Medicine and Science, Rochester, Minnesota, USA; 3 Brown University, Providence, Rhode Island, USA; 4 Tulane School of Medicine, New Orleans, Louisiana, USA; 5 Federal University of Sao Paulo (Unifesp), Sao Paulo, Brazil; 6 University of Ghent, Ghent, Belgium; 7 Cleveland Clinic, Cleveland, Ohio, USA; 8 University of Illinois, Chicago, Illinois, USA

The possibility that a lung disease is linked to liver disease was first recognized in the 1880s by German physician M. Flückiger, in the context of a young woman with liver cirrhosis, cyanosis, and digital extremity clubbing (Fluckiger 1884). The liver and lungs interact closely to regulate key physiological processes to maintain homeostasis. The hepatic and pulmonary circulation are integrally connected, with hepatic blood draining into the inferior vena cava that is then directed to the right heart and pulmonary artery (Lautt 2009).

The liver secretes a multitude of important factors that play essential regulatory roles in metabolic, immunologic, and hormonal processes. A key pillar of normal liver–lung communication is the secretion of hepatic proteins (“hepatokines”) alongside resident and recruited hepatic immune cell populations (such as macrophages) that regulate pulmonary immune cell proliferation, differentiation, and functioning (Zhao et al. 2023). This is perhaps best observed in the setting of bacterial pneumonia, where the pulmonary acute phase immune reaction is dependent upon normal liver function, specifically appropriate production and secretion of hepatic-derived proteins (Hilliard et al. 2015). The liver also plays an important role in nutrient absorption, energy metabolism, filtering of bacteria and endotoxins, and drug detoxification, all of which require normal hepatic function and adequate flow from the portal circulation (Lombardi et al. 2024).

Despite a growing appreciation for the importance of liver–lung communication in maintaining human health, little is known about the role the liver–lung axis plays in pulmonary disease. Major questions remain regarding the underlying pathophysiologic mechanisms that mediate this crucial interorgan communication network (Figure 1).

It is now established that portal hypertension and portosystemic shunting play a central role in mediating liver–lung communication in pulmonary disease. In the normal state, portal vein blood flows predominantly through the liver for processing, while a reduced portion bypasses the liver and drains directly into the systemic circulation (so-called portosystemic shunting). However, in portal hypertension, this portosystemic shunting increases, preventing hepatic metabolism or inactivation of gut-derived antigens, toxins, microbial products, vasoactive mediators, inflammatory cytokines, and other potentially dangerous substances (Silva et al. 2025). Net transfer of these substances from the portal to pulmonary circulation may initiate or potentiate parenchymal and vascular remodeling, manifesting in pulmonary disease. Additionally, liver cirrhosis-associated hyperdynamic circulation can lead to shear stress, damaging pulmonary vascular integrity.

Research to date has focused on pulmonary vascular complications of liver disease, but there is also an emerging literature on the liver as an important modulator of the pulmonary vasculature in the absence of clinical liver disease. As intrapulmonary vascular dilation is a hallmark of hepatopulmonary syndrome (HPS), factors implicated in disease pathogenesis include pro-angiogenic proteins such as vascular endothelial growth factor, inflammation from bacterial translocation and endotoxemia, and altered bile acid levels enhancing angiogenesis and vascular remodeling (Raevens et al. 2022). A deficiency of the vascular quiescence factor bone morphogenetic protein type 9 (BMP9) has also been linked to HPS in both animal models and human epidemiologic data (Robert et al. 2024). Unfortunately, treatment targeted at pro-angiogenic proteins has been unsuccessful in ameliorating the burden of pulmonary vascular disease. Treatment against other potentially causative factors remains in early development. Liver transplantation remains the only effective treatment for HPS (Kawut et al. 2019).

Molecular characterization of portopulmonary hypertension (PoPH) remains equally rudimentary. Portal hypertension is known to be a requirement for the development of PoPH, and disease pathogenesis involves similar mechanisms as HPS (pro-angiogenic factors including BMP9, systemic inflammation, sex hormone signaling, pulmonary vascular endothelial dysfunction, etc.). However, the specific mediators of liver–lung communication in PoPH remain elusive (Jose et al. 2023). Currently, treatment for PoPH centers around consideration of liver transplantation and use of pulmonary arterial hypertension (PAH) therapies (Del Valle and DuBrock 2021), despite PoPH being an exclusion criterion for most PAH therapeutic trials. Although the liver–lung axis is clearly implicated in pulmonary vascular disease pathogenesis, substantial fundamental gaps in knowledge remain, including the precise identity of the circulating factors responsible for the development of pulmonary vascular diseases due to liver disease (PoPH and HPS). It remains unclear if these diseases stem from a failure to clear damaging substances, a deficiency of protective compounds, or both (Krowka 2020).

The liver–lung axis has also been implicated more broadly in the pathogenesis of other forms of PAH. Among patients with PAH, elevated right ventricular pressures often result in hepatic congestion and dysfunction. Furthermore, liver injury has been linked to worse PAH survival in clinical studies, even after adjustment for PAH severity and other confounders (Scott et al. 2024). Recent evidence suggests that this relationship is bi-directional, with hepatic injury releasing vasoactive peptides and altering circulating levels of bile acids and inflammatory mediators, that may themselves establish or accelerate pulmonary vascular disease (Singh et al. 2025). There are also specific sub-types of PAH where the liver–lung axis is specifically stressed, namely schistosomiasis-associated PAH. Infection by the schistosoma parasitic flatworm (which matures in the liver and releases parasitic eggs into the portal and mesenteric circulation) results in hepatosplenic schistosomiasis with hepatosplenomegaly, liver fibrosis, and portal hypertension. In certain individuals, it is postulated that a combination of several factors (mechanical obstruction of pulmonary blood flow by portosystemic embolization of parasitic eggs, vascular inflammatory response to pulmonary egg deposition, dysregulated hepatic function by parasitic infection, portal hypertension and increased pulmonary vascular shear stress, and/or dysregulated immune response secondary to parasitic infection) results in vascular remodeling and the development of PAH (Sibomana et al. 2020). However, the precise factors responsible for bidirectional liver–lung communication in different forms of PAH remain undefined.

The liver–lung axis has not only been implicated in pulmonary vascular disease, but there is mounting evidence of its relevance in pneumonia and acute lung injury. As detailed earlier, intact liver–lung communication is necessary for host defense against bacterial pathogens invading the pulmonary system (Hilliard et al. 2015). Additionally, hepatic function is an important determinant of outcomes in pneumonia and acute respiratory distress syndrome (ARDS), supporting an interdependence between the liver and lungs. Epidemiologic studies suggest that liver cirrhosis is independently and significantly associated with mortality in ARDS, even after adjustment for confounders such as critical illness severity. Recent work suggests normal liver function also exerts a protective effect on lung function, and adequate hepatic reserve is not only beneficial, but necessary, for proper recovery from various forms of lung injury (Herrero et al. 2020). The precise mechanisms for this effect are unclear but are theorized to include failed hepatic clearance of bacteremia and endotoxemia, hepatic injury releasing pro-inflammatory mediators that activate pulmonary macrophages, and the release of procoagulant factors into circulation by hepatic monocytes stimulating pulmonary vascular thrombosis and cytokine release. Collectively these events result in disruption of the normal acute phase response and uncontrolled pulmonary parenchymal inflammation and remodeling.

Despite a growing acceptance that liver–lung communication plays an important role in driving pulmonary disease pathogenesis, fundamental questions persist (Krowka 2020). Characterization of the liver–lung axis is still nascent, and the precise mechanisms and circulating factors responsible for mediating liver–lung crosstalk and driving cardiopulmonary disease remain elusive. Despite liver transplantation being the definitive therapy for HPS, it has highly variable effects on PoPH, and the reason for this discrepancy is unclear. The optimal treatment strategy for pulmonary diseases where liver disease is implicated continues to evolve, and it is unclear if therapy directed towards hepatic dysfunction has benefits in these diseases. Portal hypertension appears to be a common theme across different pulmonary diseases, but whether this has mechanistic relevance remains unsettled. Finally, whether there are additional pulmonary diseases where the liver–lung axis (and possibly portal hypertension) plays a fundamental role remains an open question.

This *Call for Papers* for *Comprehensive Physiology* seeks to address these compelling issues, focusing further attention on the importance of liver–lung communication in respiratory diseases in an effort to stimulate cross-disciplinary collaborative work and encourage a coordinated effort to move the field forward. In service to this overarching aim, we are casting a wide net. All manner of submissions from basic scientists, as well as translational and clinical researchers, are welcome, including original research, editorial and commentary pieces, perspectives, and white papers. All aspects of possible liver–lung communication in pulmonary disease are sought, with a particular interest in work that examines angiogenesis, vascular remodeling, and inflammation as possible mediators of cross-organ communication. To foster collaboration, and in recognition of the wide-reaching impact that liver–lung communication has on human health and disease, the best work is eagerly sought regardless of medical specialty, level of training, or scientific discipline. By centralizing, aggregating, and integrating this knowledge in the context of this special issue, we hope to gain a deeper understanding of the liver–lung axis and identify promising new avenues of scientific inquiry that lay foundations for novel approaches to diagnosis, management, and treatment of pulmonary disease.

## Figures and Tables

**FIGURE 1 |:**
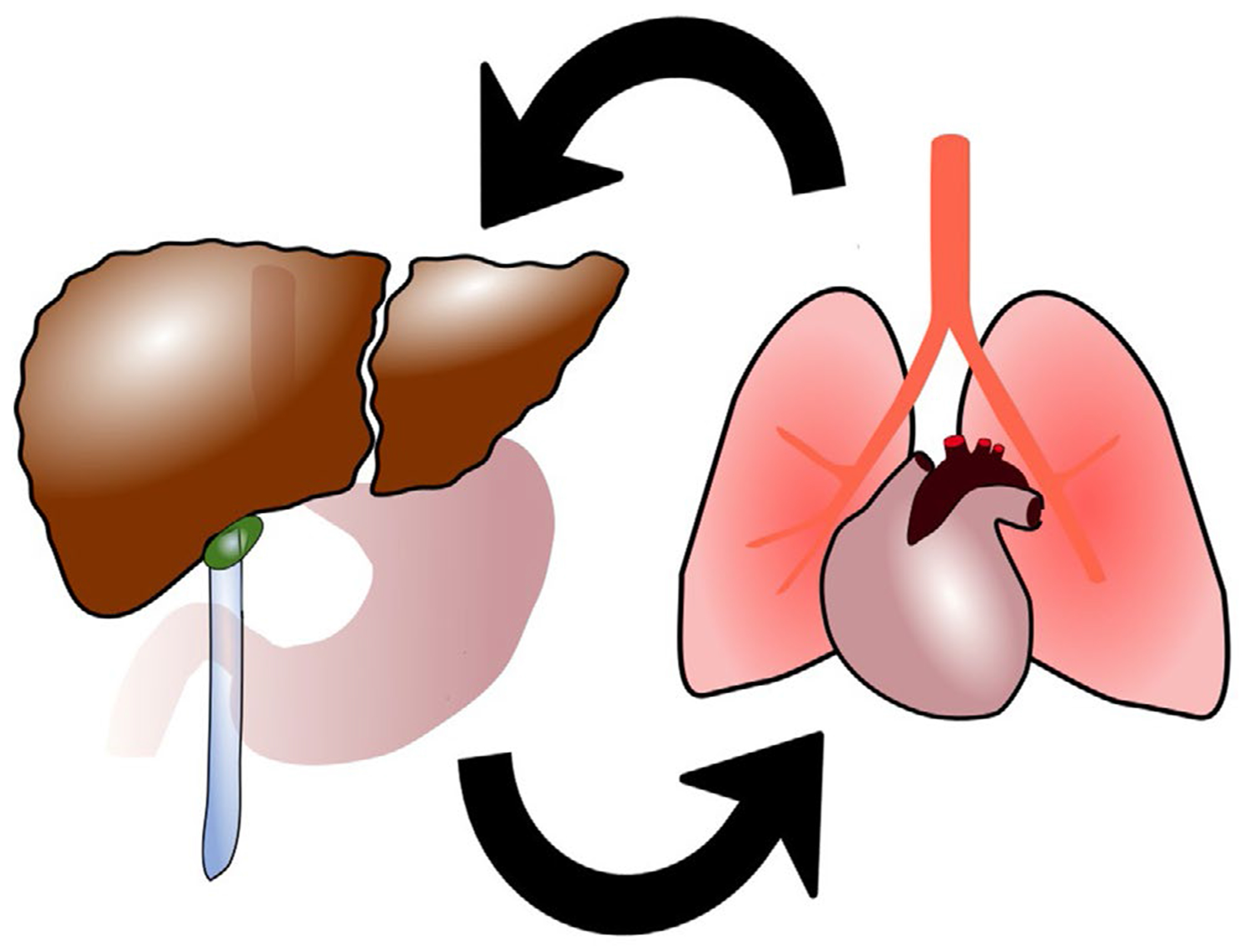
The “liver–lung” axis: Bi-directional communication between liver and lungs plays an important role in driving pulmonary disease pathogenesis.

## Data Availability

Data sharing not applicable to this article as no datasets were generated or analyzed during the current study.
